# Bibliometric Analysis of Ferroptosis in Stroke From 2013 to 2021

**DOI:** 10.3389/fphar.2021.817364

**Published:** 2022-02-21

**Authors:** Yuhua Chen, Tianlin Long, Quanhua Xu, Chi Zhang

**Affiliations:** ^1^ Department of Central Laboratory, Xi’an Peihua University, Xi’an, China; ^2^ Department of Neurosurgery, Bijie Traditional Chinese Medicine Hospital, Bijie, China; ^3^ Department of Neurosurgery, Xiangya Hospital, Central South University, Changsha, China; ^4^ The Institute of Skull Base Surgery and Neurooncology at Hunan Province, Changsha, China

**Keywords:** bibliometric analysis, Web of Science, stroke, ferroptosis, programmed cell death

## Abstract

**Background:** Stroke is a major cause of long-term disability and death, but the clinical therapeutic strategy for stroke is limited and more research must be conducted to explore the possible avenues for stroke treatment and recovery. Since ferroptosis is defined, its role in the body has become the focus of attention and discussion, including in stroke.

**Methods:** In this work, we aim to systematically discuss the “ferroptosis in stroke” research by bibliometric analysis. Documents were retrieved from the Web of Science Core Collection database on October 30, 2021. Statistical analysis and visualization analysis were conducted by the VOSviewer 1.6.15.

**Results:** Ninety-nine documents were identified for bibliometric analysis. Research on “ferroptosis in stroke” has been rapidly developing and has remained the focus of many scholars and organizations in the last few years, but the Chinese groups in this field still lacked collaboration with others. Documents and citation analysis suggested that Rajiv R. Ratan and Brent R. Stockwell are active researchers, and the research by Qingzhang Tuo, Ishraq Alim, and Qian Li are more important drivers in the development of the field. Keywords associated with lipid peroxidation, ferroptosis, iron, oxidative stress, and cell death had high frequency, but apoptosis, necroptosis, pyroptosis, and autophagy had scant research, and there may be more research ideas in the future by scholars.

**Conclusion:** Further exploration of the mechanisms of crosstalk between ferroptosis and other programmed cell death may improve clinical applications and therapeutic effects against stroke. Scholars will also continue to pay attention to and be interested in the hot topic “ferroptosis in stroke”, to produce more exciting results and provide new insights into the bottleneck of stroke treatment.

## Background

With about 3.0 million new cases occurring every year, stroke is a primary reason of disability and death in China and the United States (Wu et al., 2019; Barthels and Das, 2020). The burden is expected to increase further due to population aging, continued high prevalence of risk factors such as hypertension, and poor management. Although overall access to health services has improved, access to specialist stroke cure and care is patchy across regions and particularly uneven in backward areas (Wu et al., 2019; Zhang et al., 2020). Research has shown that people with hypertension, obesity, and diabetes have a higher incidence of stroke (Barthels and Das, 2020). The vast majority of strokes fall into two categories, hemorrhagic stroke (HS) induced by ruptured brain blood vessels and ischemic stroke (IS) caused by blocked brain arteries, both of which lead to local hypoxia and brain tissue damage (Campbell et al., 2019; Barthels and Das, 2020). Currently, tissue plasminogen activator (tPA), a powerful thrombolysis for the dissolution of acute thromboembolism, is the only ischemic stroke drug approved by the Food and Drug Administration (Kuo et al., 2020). But the tPA has a strict time window for treatment. Stroke patients must receive tPA within 3 h of the onset of stroke symptoms, but no longer than 4.5 h, and if the treatment window is exceeded, it may lead to hemorrhagic transformation, which can cause additional damage to the brain (Hughes et al., 2021). Routine prophylactic drug interventions, such as anticoagulants and blood pressure-lowering and cholesterol-lowering drugs, are necessary for people who have experienced stroke because they are at increased risk for a second stroke occurring immediately after the first stroke (Barthels and Das, 2020). Dismayingly, there is still no effective treatment for HS (Yin et al., 2021). Overall, the clinical therapeutic strategy for stroke is limited, and more research must be conducted to explore possible avenues for stroke treatment and recovery.

Ferroptosis is a programmed cell death (PCD) characterized by iron-dependent lipid peroxidation, including impaired intracellular cysteine uptake, glutathione depletion, membrane damage, and damage-related molecule release (Galluzzi et al., 2018; Tang and Kroemer, 2020; Hassannia et al., 2021; Wu et al., 2021). The term “ferroptosis” was coined by Scott J Dixon in 2012 and used to describe a type of cell death caused by erastin and also suppressed by lipophilic antioxidants or iron chelators such as ferrostatin-1 (Dixon et al., 2012). Recent studies have shown that the cell phenotype and molecular events of ferroptosis are different from apoptosis, autophagy, necroptosis, pyroptosis, and other PCDs (Galluzzi et al., 2018; Zhou et al., 2020). PCD is crucial to the occurrence and development of multiple diseases, including dysgenopathy, immune system diseases, central nervous system (CNS) disease, and cancer (Gibellini and Moro, 2021; Kist and Vucic, 2021; Moujalled et al., 2021). Moreover, common molecular events involved in ferroptosis are logically linked to the occurrence and development of many diseases, such as oxidative stress and abnormal iron, glutamate, and lipid metabolism (Stockwell et al., 2020; Plascencia-Villa and Perry, 2021; Yu et al., 2021). Ferroptosis involves nerve disorders (David et al., 2021), such as Alzheimer’s disease (AD) (Plascencia-Villa and Perry, 2021), Parkinson’s disease (PD) (Mahoney-Sánchez et al., 2021, spinal cord injury (SCI) (Ge et al., 2021), traumatic brain injury (TBI) (Rui et al., 2020), stroke (Alim et al., 2019; Zhou et al., 2020), and depression (Cao et al., 2021). The novel findings provide potential strategies for the clinical treatment and prognosis of stroke, such as targeting ferroptosis. However, to the best of our knowledge, the objective and overall reports on the publishing trend, powerful research, institutions and their collaborations, and the hotspots of “ferroptosis in stroke” are lacking.

In this work, we aim to systematically discuss the “ferroptosis in stroke” research from 2013 to 2021 by bibliometric analysis. The bibliometric analysis combines mathematical and statistical methods with data visualization (Chen G. et al., 2021), to present the annual publications, countries/regions, institutions, journals, authors, and co-citation; to evaluate global patterns of collaboration between authors, institutions, and countries; and to determine the study trend and hotspot in “ferroptosis in stroke”.

## Data and Method

### Data Collection

The keywords of ferroptosis and stroke were indexed in the Web of Science Core Collection (WOSCC). Articles from 2013 to 2021 (deadline October 30, 2021) were retrieved, and search themes were as follows: “TS=(ferroptosis) OR TS=(ferroptotic)”, AND “TS=(stroke) OR TS=(cerebrovascular accident) OR TS=(cerebrovascular apoplexy)”, AND Language: English, AND Reference Type: Article OR Review”. A total of 99 references were chosen and then used to perform a bibliometric analysis.

### Data Analysis

Firstly, through the analysis and retrieval results in WOSCC, the general information of the literature is preliminarily analyzed, including the year of publication, country, organization, journal, and author. Then, the VOSviewer 1.6.15 software was used to conduct bibliometric and visual analysis, including main author, keyword, scientific research partnership, cited analysis, and co-cited analysis. The standard tournament ranking method was used for ranking order, and Linlog/modularization was applied in the VOSviewer software.

## Results

### Publication Outputs

**FIGURE 1:**
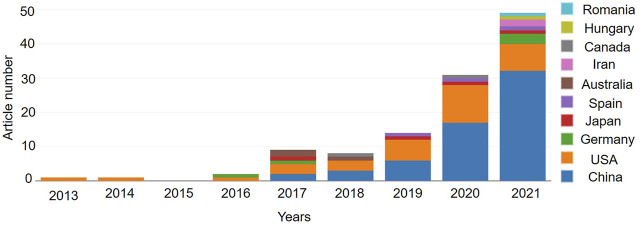
The annual publication indexed in the WOSCC from 2013 to 2021 by online bibliometric analysis. And the article number of the top 10 countries/regions with “ferroptosis in stroke” were presented.

There were 99 items on “ferroptosis in stroke” in the WOSCC from 2013 to 2021 (October 30, 2021), including 51 articles (51.52%) and 48 reviews (48.48%). The annual publication is exhibited in Figure 1. There was one publication in 2013, which subsequently increased year by year, and it was 28 in 2020 and 42 on October 30, 2021 (Figure 1). The number of publication was small, but it rose steadily rise. A total of 185 sources of funding supported the research on “ferroptosis in stroke”. The top three major sources of funding were the National Natural Science Foundation of China (frequency, 43), National Institutes Of Health (frequency, 22), and United States Department of Health Human Services (frequency, 22) Table 1.

**TABLE 1:** The top 10 funding sources.

**Ranking**	**Funding Source**	**Frequency**
1	National Natural Science Foundation of China	43
2	National Institutes of Health	22
3	United States Department of Health Human Services	22
4	NIH National Institute of Neurological Disorders Stroke	12
5	American Heart Association	6
6	Burke Foundation	6
7	Dr Miriam And Sheldon G Adelson Medical Research Foundation	6
8	European Commission	6
9	NIH National Institute on Aging	6
10	NIH National Cancer Institute	5

### Countries and Organization

**FIGURE 2:**
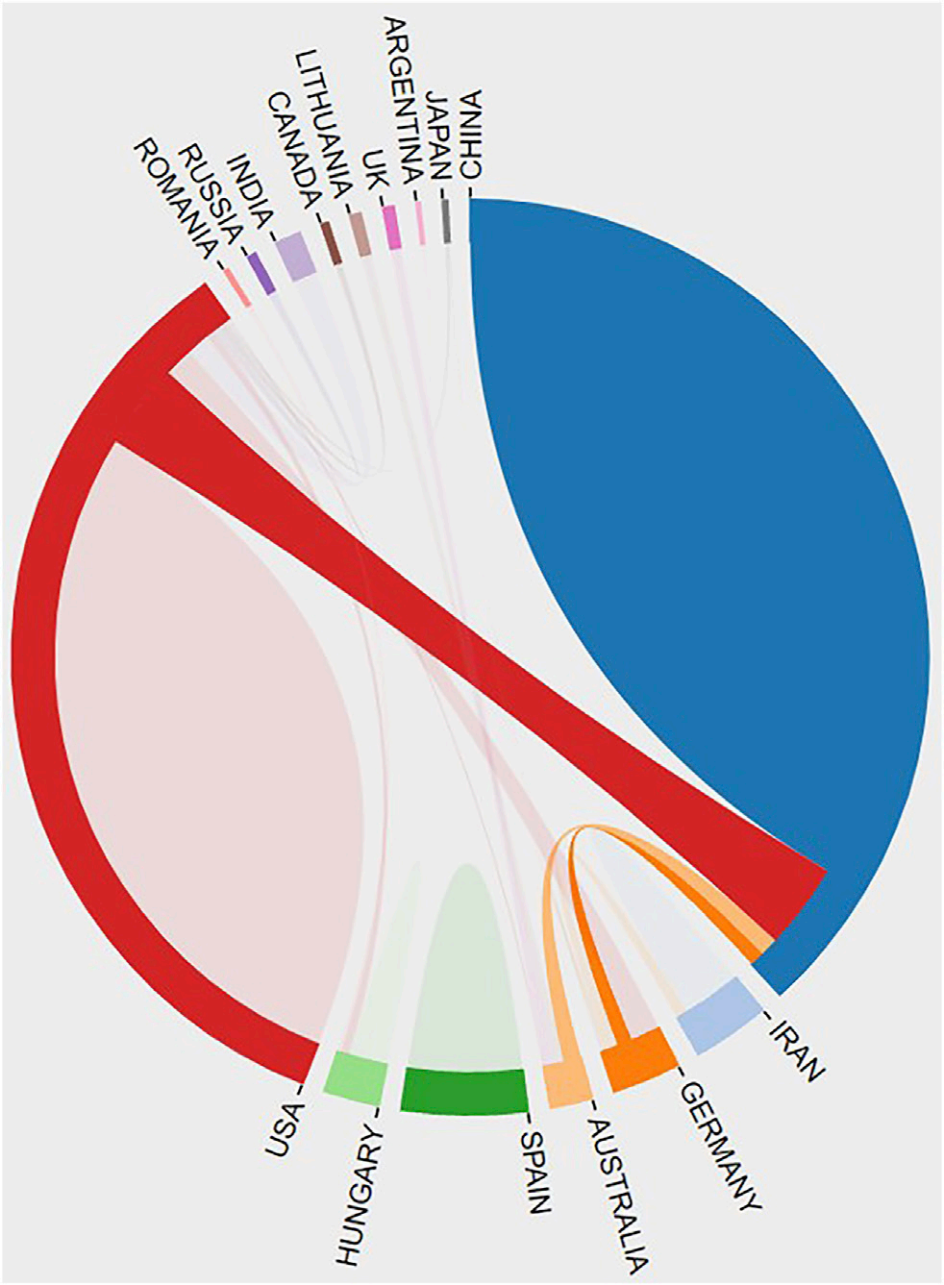
The cooperative relationship between countries was generated by the online bibliometric analysis.

**FIGURE 3:**
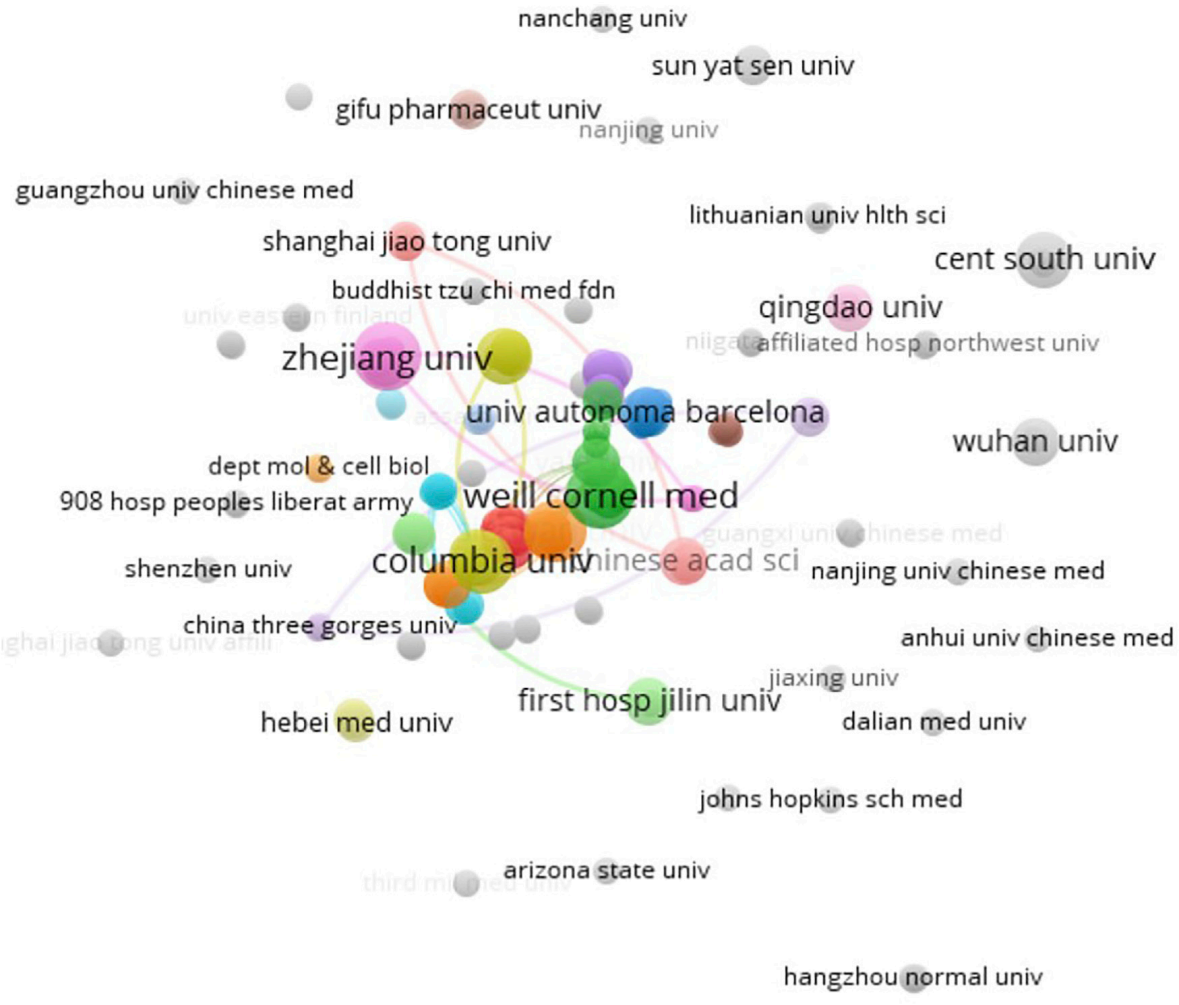
Co-author analysis of organizations with network visualization by the VOSviewer 1.6.14. Some of the 190 items in the network. The thickness of the lines indicates the strength of the relationship.

There were 15 countries and 193 organizations in the 99 documents of “ferroptosis in stroke”, which had been published in the past 8 years. China (63), the United States (38), and Germany (5) were the top three countries/regions, and early research began in the United States and China, which have published documents mainly since 2017 (Figure 1). The United States had the most citation with 2,834, but the citation of publication in China was 2,262. Cooperation on this subject has been concentrated between China and the United States, with weak cooperation between other countries (Figure 2). As shown in Table2, the top 10 institutions in terms of publications were from China (50%) and the United States (50%). The top three institutions in terms of publications were Columbia University (United States, six documents), Weill Cornell Medicine (United States, six documents), and Zhejiang University (China, six documents), but the top three institutions ranked by citations were Columbia University (1,819), Yale University (1,731), and Memorial Sloan Kettering Cancer Center (1,596) (Table 2). Cooperation between institutions is presented in Figure 3. The top organizations showed extensive relationships with others (Figure 3), but some gray circles indicated that the institutions were isolated. The data suggest that the top institutions with “ferroptosis in stroke” research lack cooperation with each other; in particular, the Chinese institutions should improve cooperation with their counterparts.

**TABLE 2:** Top 10 most productive organizations.

**Rank**	**Organizations**	**Country**	**Documents**	**Citations**
1	Columbia University	United States	6	1819
2	Weill Cornell Medicine	United States	6	459
3	Zhejiang University	China	6	27
4	Sichuan University	China	5	245
5	Memorial Sloan Kettering Cancer Center	United States	4	1596
6	Johns Hopkins University	United States	4	443
7	Central South University	China	4	24
8	Jilin University	China	4	24
9	Yale University	United States	3	1731
10	Harbin Medical University	China	3	200

### Journals

There were 72 journals with documents on “ferroptosis in stroke”. In Table 4, the top 10 journals were shown to have published about 34.34% of documents (34/99). *Frontiers in Cellular Neuroscience* was the most active journal, followed by *Frontiers in Neuroscience*, *Frontiers in Cell and Developmental Biology*, *Frontiers in Pharmacology*, *Life Sciences*, *Frontiers in Neurology*, *Cell*, *Cellular and Molecular Life Sciences*, *Pharmacology and Pharmacy*, and *Cell Chemical Biology*. All of the journals had a higher level of JCR partition (70% Q1 and 30% Q2), and *Cell Chemical Biology* had a minimum IF of 4.003 (Q2) (Table 3). Encouragingly, one article and one review were published in the *Cell* journal (Q1, 41.584). The publication of these articles in the corresponding high-level journals is enough to indicate that “ferroptosis in stroke” is exciting and interesting.

**TABLE 3:** Top 10 journals with the largest number of publications.

Rank	Journals	Documents	2020 Impact Factor	2020 JCR Partition
1	*Frontiers in Cellular Neuroscience*	8	5.505	Q1
2	*Frontiers in Neuroscience*	4	4.677	Q2
3	*Frontiers in Cell and Developmental Biology*	3	6.684	Q1/Q2
4	*Frontiers in Pharmacology*	3	5.811	Q1
5	*Life Sciences*	3	5.037	Q1/Q2
6	*Frontiers in Neurology*	3	4.003	Q2
7	*Cell*	2	41.584	Q1
8	*Cellular and Molecular Life Sciences*	2	9.261	Q1
9	*Pharmacology and Pharmacy*	2	7.658	Q1
10	*Cell Chemical Biology*	2	8.116	Q2

### Authors

**FIGURE 4:**
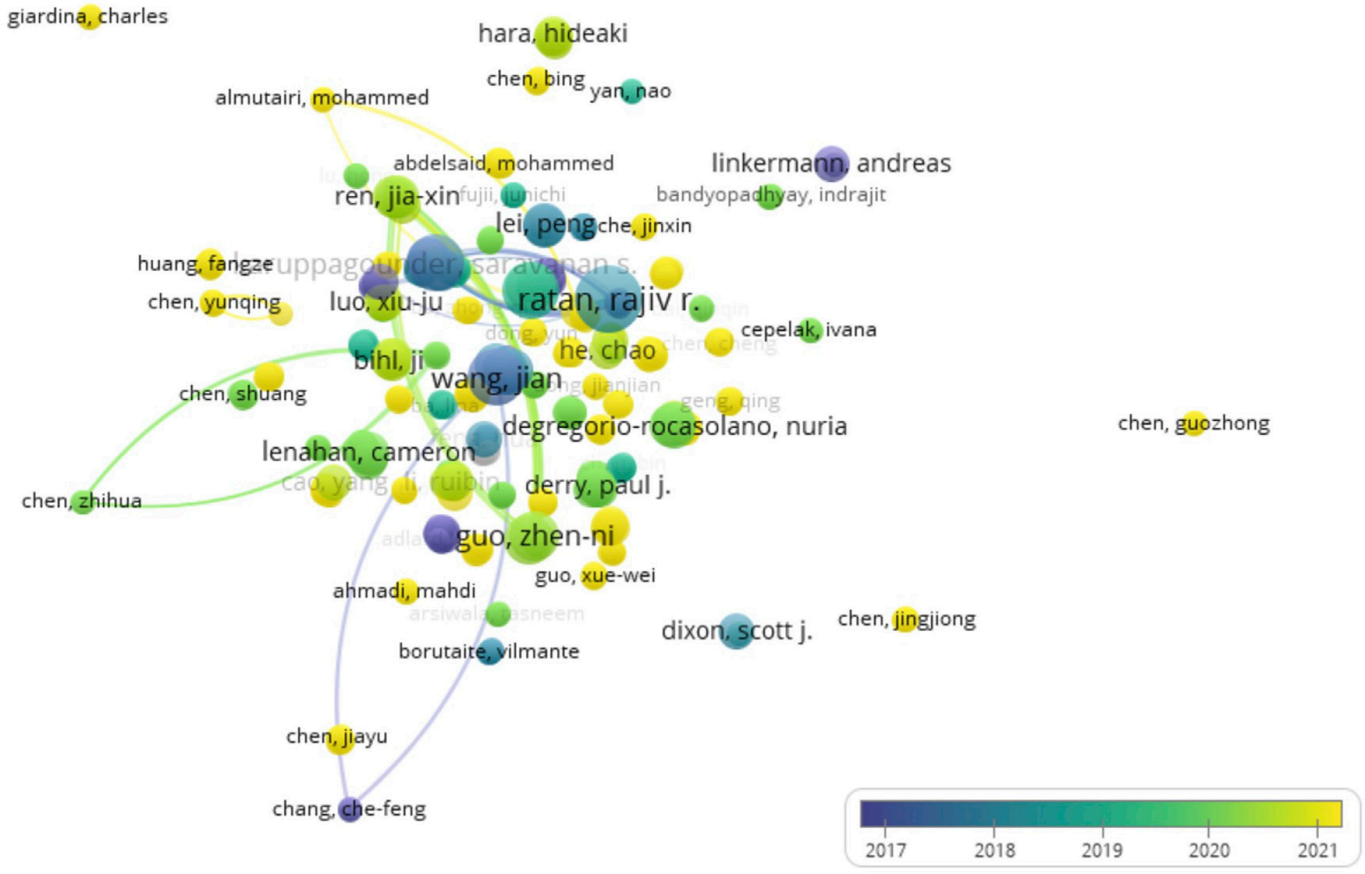
Co-occurrence analysis of authors. The author–date (656 items) was generated, and the color means the average published year.

A total of 656 authors drafted the 99 documents in “ferroptosis in stroke”. Rajiv R. Ratan (Weill Cornell Medicine) contributed seven documents and ranked first (531 citations), followed by Saravanan S. Karuppagounder (Weill Cornell Medicine), Jian Wang (Johns Hopkins University), Brent R. Stockwell (Columbia University), and Zhenni Guo (Jilin University). Seventy percent of authors were from China (Table 4). In a co-authorship map, yellow indicated that many scholars have only recently begun to work on this topic (Figure 4). Some researchers were also scattered independently with other active scholars, and Rajiv R. Ratan, Jian Wang, and Brent R. Stockwell were the center, but they had not been able to reach all groups (Figure 4). The data suggest that the active authors on “ferroptosis in stroke” still lack collaboration with other scholars.

**TABLE 4:** Top 10 active authors with most documents.

**Rank**	**Authors**	**Organizations**	**Documents**	**Citations**
1	Rajiv R Ratan	Weill Cornell Medicine	7	531
2	Jian Wang	Johns Hopkins University	5	477
3	Saravanan S Karuppagounder	Weill Cornell Medicine	5	492
4	Brent R Stockwell	Columbia University	4	441
5	Zhen-Ni Guo	Jilin University	4	24
6	Xuejun Jiang	Memorial Sloan Kettering Cancer Center	4	218
7	Peng Lei	Sichuan University	4	243
8	Qing-Zhang Tuo	Sichuan University	4	243
9	Xiu-Li Yan	Jilin University	4	24
10	Yi Yang	Jilin University	4	24

### Citations

The top 10 highly cited references and the citation analysis of documents on “ferroptosis in stroke” are shown in Tables 5, 6. “Ferroptosis: an iron-dependent form of nonapoptotic cell death” was the most cited reference in “ferroptosis in stroke” (Table 5), as Dixon et al. (2012) firstly came up with the concept of ferroptosis. Two reviews of Brent R. Stockwell were included in the highly cited references: “Ferroptosis: a regulated cell death nexus linking metabolism, redox biology, and disease” with 1,378 citations and “Emerging mechanisms and disease relevance of ferroptosis” with 84 citations (Table 6). Four of the documents appeared on both lists: “Ferroptosis: a regulated cell death nexus linking metabolism, redox biology, and disease” (review), “Tau-mediated iron export prevents ferroptotic damage after ischemic stroke” (article), “Selenium drives a transcriptional adaptive program to block ferroptosis and treat stroke” (article), and “Inhibition of neuronal ferroptosis protects hemorrhagic brain” (article), which were respectively completed by the team of Brent R. Stockwell, Qingzhang Tuo, Ishraq Alim, and Qian Li. The data suggest that Brent R. Stockwell is very interested in the research on “ferroptosis in stroke”, but the research of Qingzhang Tuo, Ishraq Alim, and Qian Li are more important drivers in the development of the field, and articles of “ferroptosis in stroke” with a high number of citations have been widely accepted and have inspired recent researches, and recent studies should improve scale and breakthrough.

**TABLE 5:** Top 10 co-citation of cited references on “ferroptosis in stroke”.

**Rank**	**Title**	**Type**	**First author**	**Source**	**Publication year**	**Total citations**
1	Ferroptosis: an iron-dependent form of nonapoptotic cell death	Review	SJ Dixon	Cell	2012	82
2	Regulation of ferroptotic cancer cell death by GPX4	Article	WS Yang	Cell	2014	51
3	Ferroptosis: a regulated cell death nexus linking metabolism, redox biology, and disease	Review	BR Stockwell	Cell	2017	50
4	Inactivation of the ferroptosis regulator Gpx4 triggers acute renal failure in mice	Article	JPF Angeli	Nat Cell Biol	2014	44
5	Tau-mediated iron export prevents ferroptotic damage after ischemic stroke	Article	QZ Tuo	Mol Psychiatr	2017	42
6	Selenium drives a transcriptional adaptive program to block ferroptosis and treat stroke	Article	I Alim	Cell	2019	42
7	Inhibition of neuronal ferroptosis protects hemorrhagic brain	Article	Q Li	JCI Insight	2017	39
8	Ferroptosis: process and function	Review	Y Xie	Cell Death Differ	2016	39
9	Glutaminolysis and transferrin regulate ferroptosis	Article	MH Gao	Mol Cell	2015	39
10	ACSL4 dictates ferroptosis sensitivity by shaping cellular lipid composition	Article	S Doll	Nat Chem Biol	2017	37

**TABLE 6:** Top 10 citation analysis of documents on “ferroptosis in stroke”.

**Rank**	**Title**	**Type**	**First author**	**Source**	**Publication year**	**Total citations**
1	Ferroptosis: a regulated cell death nexus linking metabolism, redox biology, and disease	Review	BR Stockwell	Cell	2017	1378
2	Neuronal cell death	Review	M Fricker	Physiol Rev	2018	275
3	Inhibition of neuronal ferroptosis protects hemorrhagic brain		Q Li	JCI Insight	2017	223
4	Neuronal death after hemorrhagic stroke *in vitro* and *in vivo* shares features of ferroptosis and necroptosis	Article	M Zille	Stroke	2017	192
5	Tau-mediated iron export prevents ferroptotic damage after ischemic stroke	Article	QZ Tuo	Mol Psychiatr	2017	183
6	Selenium drives a transcriptional adaptive program to block ferroptosis and treat stroke	Article	I Alim	Cell	2019	161
7	Ischemia-induced ACSL4 activation contributes to ferroptosis-mediated tissue injury in intestinal ischemia/reperfusion	Article	Y Li	Cell Death Differ	2019	132
8	Ferroptosis and its role in diverse brain diseases	Review	A Weiland	Mol Neurobiol	2019	94
9	Ferroptosis: mechanisms, biology and role in disease	Review	XJ Jiang	Nat Rev Mol Cell Biol	2021	93
10	Emerging mechanisms and disease relevance of ferroptosis	Review	BR Stockwell	Trends Cell Biol	2020	84

**FIGURE 5:**
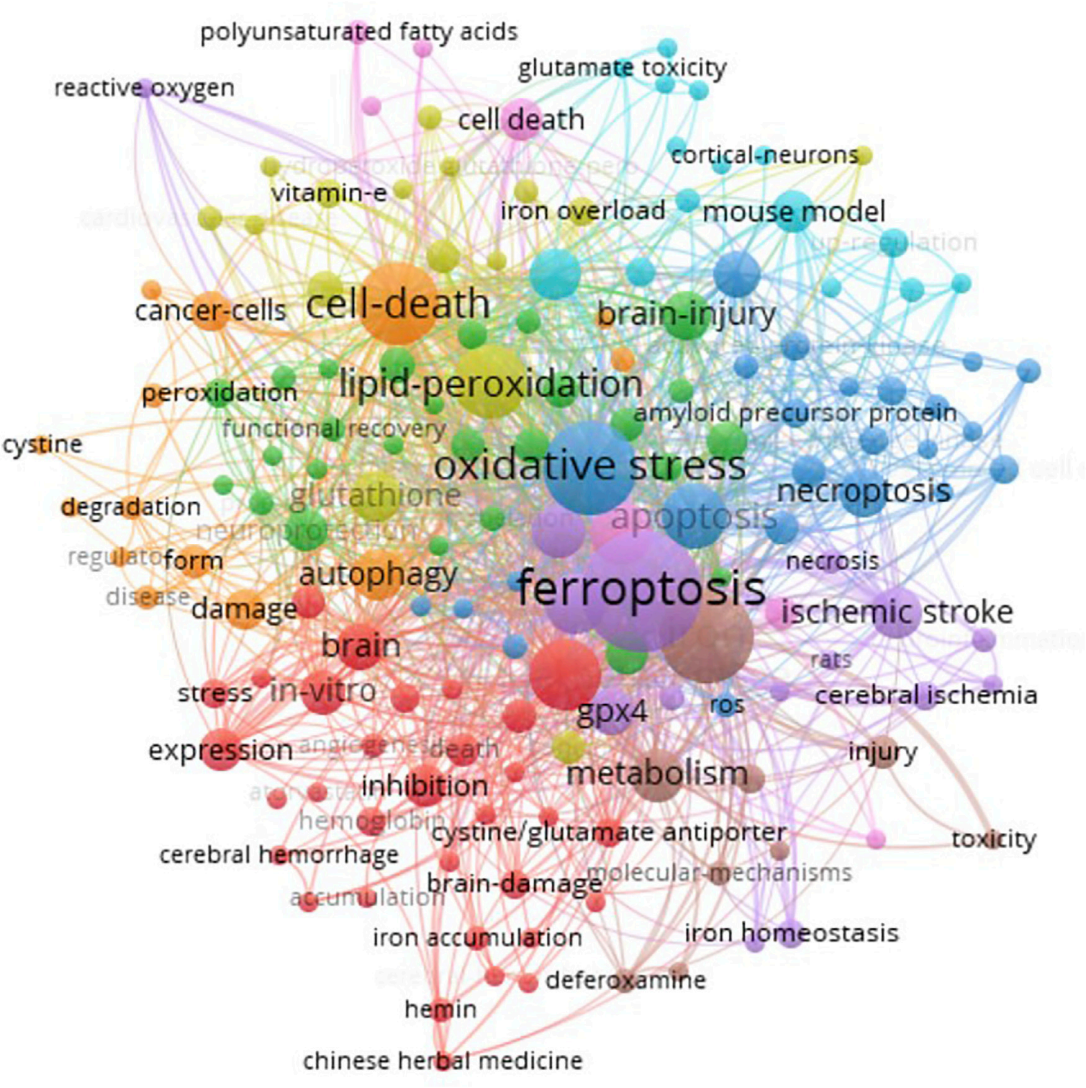
Co-occurrence analysis of author keywords. Two minimum number of occurrences of a keyword, and 158 meet the threshold of the 554 keywords. The network visualization of the keywords (158 items) was conducted.

**FIGURE 6:**
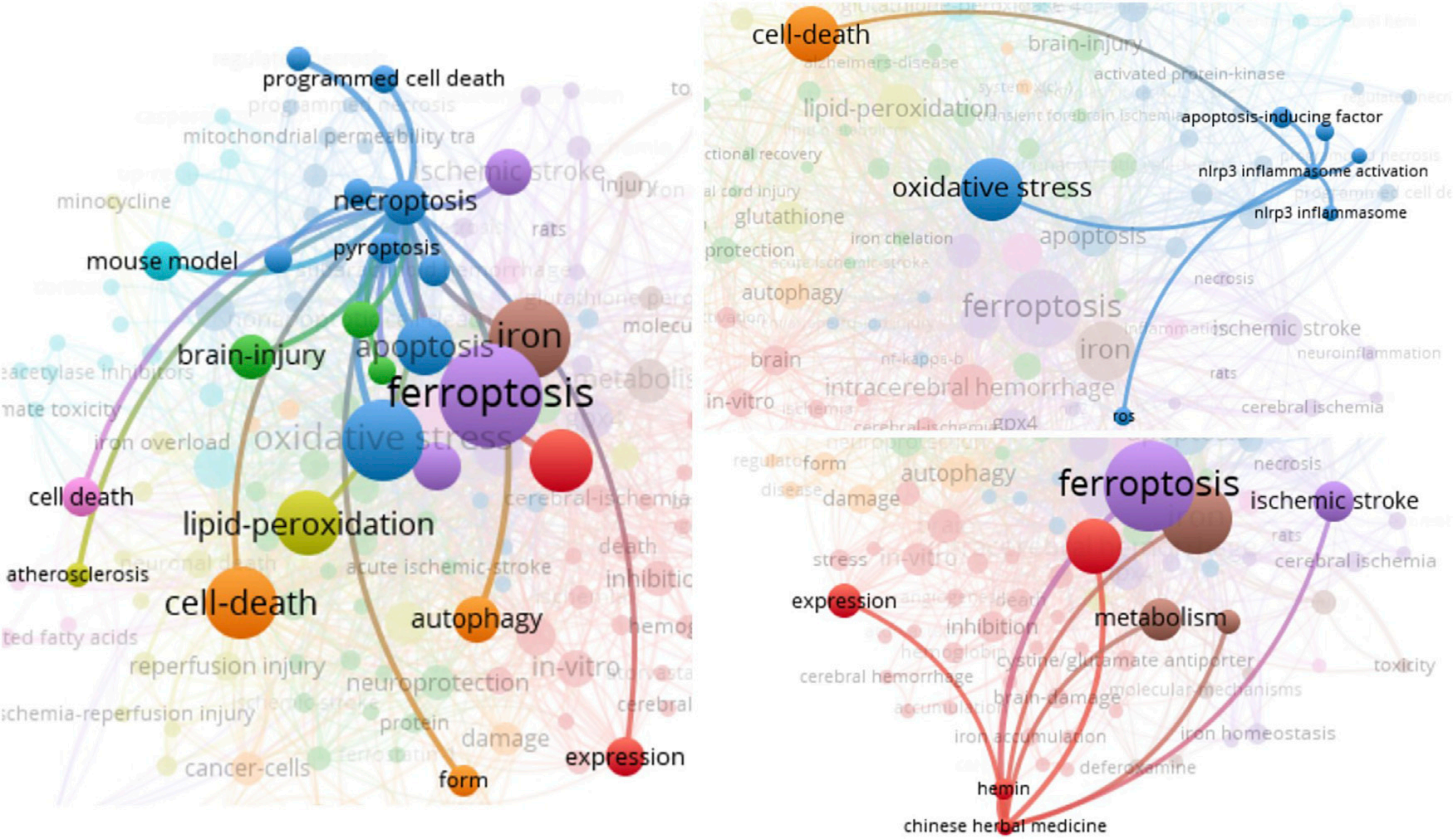
The crosstalk of author keywords necroptosis, NLRP3 inflammasome activation, and Chinese herbal medicine was presented.

Figure 5 showed the keyword subnetwork and its clustering graph, and the circle size represented the number of occurrences of keywords. The top six keywords in terms of occurrence were “ferroptosis”, “oxidative stress”, “iron”, “cell death”, “lipid peroxidation”, and “intracerebral hemorrhage” (Figure 5). In the overlay visualization (Figure 6), the keywords apoptosis, necroptosis, pyroptosis, autophagy, NLRP3 inflammasome activation, and Chinese herbal medicine were are also related to ferroptosis but had few research.

## Discussion

In the last few years, research on “ferroptosis in stroke” has been rapidly developing and remained the focus of scholars and organizations. Some countries, organizations, and scholars collaborated on research on “ferroptosis in stroke”, but the Chinese groups in this field still lacked collaboration with others. The documents and citation analysis suggested that Rajiv R. Ratan and Brent R. Stockwell were active researchers, and the research of Qingzhang Tuo, Ishraq Alim, and Qian Li are more important drivers in the development of the field. Keywords associated with lipid peroxidation, ferroptosis, iron, oxidative stress, and cell death were highly frequent, but apoptosis, necroptosis, pyroptosis, and autophagy were used in few research, so there may be more research ideas in the future by scholars. “Ferroptosis in stroke” is an emerging research topic that will continue to produce more exciting results and provide new insights into the bottleneck of stroke treatment.

***Ferroptosis, a newly defined class of PCD in 2012 (Dixon et al., 2012), quickly became the focus of attention and discussion because of its important function in the body, and publication on this topic has increased year by year. In 2013, Louandre et al. demonstrated that sorafenib induces ferroptosis in hepatocellular carcinoma (HCC) cells, and triggering ferroptosis may improve the antitumor effect of sorafenib in HCC (Louandre et al., 2013); Henke et al. (2013) found that dysregulated ORAI1-mediated Ca^2+^ influx contributes to ferroptosis in HT22 cells; based on their previous research, Speer et al. (2013) suggested that iron chelators inhibit ferroptosis in primary neurons by targeting the HIF prolyl hydroxylases. Since then, research on “ferroptosis” and “ferroptosis in stroke” has been gradually reported.

Studies have confirmed that ferroptosis involves the failure of GPX4 activity and the metabolic processes of iron, amino acids, and lipid peroxide, which results in the accumulation of intracellular reactive oxygen species (ROS) and ferroptotic death (Stockwell et al., 2017; Wan et al., 2019; Cheng et al., 2021). Ferroptosis is mediated by nuclear factor E2-related factor 2 (NRF2) and Hippo pathways by regulating GPX4 activity (Liu et al., 2021). There are still polymolecules involved in ferroptosis, such as acyl-CoA synthetase long-chain family member 4 deferoxamine (ACSL4), ferritin, divalent metal transporter 1 (DMT1), glutathione (GSH), imidazole ketone erastin, lysophosphatidylcholine acyltransferase 3 (LPCAT3), nicotinamide adenine dinucleotide phosphate (NADPH), oxidized glutathione (GSSG), solute carrier family three member 2 (SLC3A2), transferrin, and transferrin receptor 1 (TFR1) (Liu et al., 2021; Zhang et al., 2021). With the deepening of research, the regulatory network of ferroptosis in stroke is gradually revealed (Bai et al., 2020; DeGregorio-Rocasolano, et al., 2019; Zille et al., 2017). Ferritin reduces robust ROS production and GSH consumption; its decrease is necessary for cerebral ischemia-induced hippocampal neuronal ferroptosis through p53 and SLC7A11 in middle cerebral artery occlusion (MCAO) rats (Chen W. et al., 2021), and mice lacking mitochondrial ferritin show graver brain injury and neurological deficits, accompanied by typical ferroptotic event after cerebral ischemia/reperfusion (I/R) (Wang et al., 2021). ACSL4 enhances ischemic stroke by increasing ferroptosis-induced brain damage and neuroinflammation while inhibiting ACSL4, which promotes the recovery of neurological function following stroke (Li et al., 2019; Chen J. et al., 2021; Cui et al., 2021). Tuo et al. (2017) demonstrated that tau suppression reduces MCAO-induced ferroptosis and influences ischemic stroke outcome. Nuclear receptor coactivator 4 (NCOA4) facilitates ferritinophagy-mediated ferroptosis, and NCOA4 deletion protects neurons from ferritinophagy-mediated ferroptosis after ischemic stroke (Li C. et al., 2021). Guo et al. (2021) hypothesized that PIEZO1 involves cerebral I/R injury *via* ferroptosis regulation. In addition, noncoding RNAs are also involved in ferroptosis-mediated stroke. The lncRNA-MEG3/p53 signaling pathway mediates ferroptosis of rat brain microvascular endothelial cells *via* regulation of the GPX4 transcription and expression (Chen C. et al., 2021). The level of lncRNA-PVT1 is upregulated and the miR-214 level is downregulated in the plasma of acute ischemic stroke patients, and PVT1 involves ferroptosis *via* miR-214-mediated TFR1 and TP53 levels in brain I/R (Lu et al., 2020). By the bioinformatic analysis, three ferroptosis-related biomarkers are found as potential diagnostic biomarkers for ischemic stroke, namely, PTGS2, MAP1LC3B, and TLR4, which are upregulated in ischemic stroke and provide more evidence about the important role of ferroptosis (Chen G. et al., 2021). Therefore, extensive evidence suggests that ferroptosis is one of the key pathological mechanisms of nerve injury and neurological dysfunction after stroke, which is a potentially promising therapeutic target (Jin Y. et al., 2021; Li Y. et al., 2021).

**FIGURE 7:**
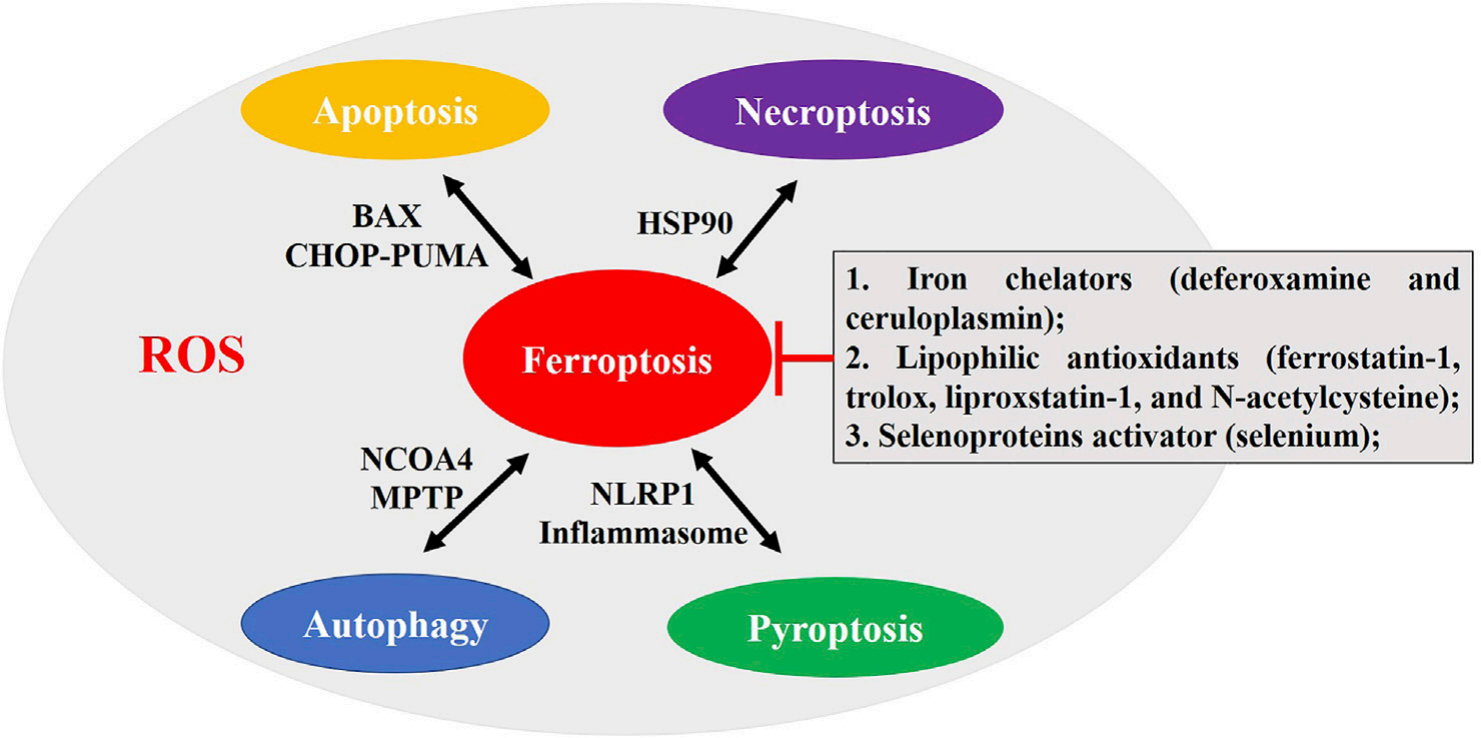
The crosstalk between ferroptosis and autophagy, apoptosis, necroptosis, and pyroptosis. The iron chelators (deferoxamine and ceruloplasmin), lipophilic antioxidants (ferrostatin-1, trolox, liproxstatin-1, and *N*-acetylcysteine), and selenoprotein activator (selenium) can reverse ferroptosis. Mitochondrial function and ROS production may be key to the crosstalk. Some molecules participate in the crosstalk between them, including ROS; BAX; HSP90; mitochondrial permeability transition pore (MPTP); NCOA4; NACHT, LRR, and PYD domains-containing protein 1 (NLRP1) inflammasome; C/EBP-homologous protein (CHOP); and p53 upregulated modulator of apoptosis (PUMA).

Previous studies show that ferroptosis is reversed by iron chelators (deferoxamine and ceruloplasmin), lipophilic antioxidants (ferrostatin-1, trolox, liproxstatin-1, and *N*-acetylcysteine (NAC)), and selenoprotein activator (selenium) (Figure 7) (Liu et al., 2021; Zhang et al., 2021). Then, significant progress has been made in preclinical studies targeting ferroptosis in the treatment of stroke. Intracerebral hemorrhage-induced neurological defects, dysmnesia, and brain atrophy were decreased by the ferroptosis inhibitor ferrostatin-1 (Chen et al., 2019). NAC, a clinically approved redox regulatory compound containing mercaptan, prevents hemin-induced ferroptosis by inhibiting toxic lipid generated by arachidonic acid-dependent ALOX5 activity (Karuppagounder et al., 2018). Deferoxamine treatment prevents post-stroke cognitive impairment in diabetes while increasing AQP4 polarity and blood–brain barrier permeability (Abdul et al., 2021). Pyridoxal isonicotinoyl hydrazine protects mice against hemorrhage stroke, by reducing ferroptosis, including ROS production, iron accumulation, and lipid peroxidation in perihematoma (Zhang et al., 2021). Pharmacological selenium inhibits GPX4-dependent ferroptotic death, and selenome protects neurons and improves behavior following hemorrhagic stroke through regulating TFAP2c and Sp1 (Alim et al., 2019). And pretreatment with selenium compounds, such as methylselenocysteine or selenocystamine, also protects I/R neuronal ferroptosis *in vivo* (Tuo et al., 2021). Some natural products and traditional Chinese medicine-related content have also been confirmed to improve the prognosis of stroke by regulating ferroptosis. The baicalin, carthamin yellow, dauricine, (−)-epicatechin, kaempferol, and paeonol ameliorate neuronal ferroptosis *in vitro* or *in vivo* stroke models via regulating ACSL4, GPX4, TFR1, Fe^2+^, and NRF2 pathways (Chang et al., 2014; Duan et al., 2021; Guo et al., 2021; Jin Z. L. et al., 2021; Peng et al., 2021; Yuan et al., 2021), which show great potential in the treatment of stroke. Treating MCAO rats with Naotaifang (a traditional Chinese herbal medicine compound) extract involves inhibition of acute cerebral ischemia-induced neuronal ferroptosis and neurobehavioral disorder through TFR1/DMT1 and SCL7A11/GPX4 pathways (Lan et al., 2020). Electroacupuncture inhibits ferroptosis to protect against MCAO via regulating iron and oxidative stress-related protein (Li G. et al., 2021). Furthermore, the new inhibitors of ferroptosis are also being explored. Yang et al. conducted promethazine derivatives and screened a promising lead compound, 2-(1-(4-(4-methylpiperazin-1-yl)phenyl)ethyl)-10*H*-phenothiazine, as a new type of ferroptosis inhibitor, which displays favorable pharmacokinetic properties, good ability to permeate the blood–brain barrier, and excellent therapeutic effect in the ischemic stroke model (Yang et al., 2021). Keuters et al. (2021) demonstrated that the benzo[b]thiazine derivative efficiently suppresses GSH or GPX4 inhibition-induced ferroptosis in neuronal cell lines and decreases infarction volume, edema, and pro-inflammatory levels after stroke. Thus, ferroptosis inhibitors and targeting them are potential treatment options for stroke, but more direct clinical evidence remains to be explored.

The keyword analysis revealed that multiple PCDs were involved in the progression of stroke pathology, and apoptosis, necroptosis, pyroptosis, and autophagy were related to ferroptosis. Some reports show that apoptosis, autophagy, pyroptosis, and ferroptosis are present together in stroke-induced neuronal damage (Guo et al., 2021; Li et al., 2018). The crosstalk between ferroptosis and other PCDs is fascinating (Figure 7). Lee et al. (2018, 2020) showed ferroptotic and apoptotic agent interactions via the B-cell lymphoma-2 associated X (BAX)-dependent mitochondrial pathway and ER stress-mediated PERK-eIF2α-ATF4-CHOP-PUMA pathway. Studies have found that ferritinophagy-mediated ROS production contributes to ferroptosis and apoptosis (Li L. et al., 2021). Ferroptosis, an autophagic cell death process, leads to autophagy activation and consequent ferritin and NCOA4 degradation (Gao et al., 2016), and NCOA4-regulated ferritinophagy maintains ferroptosis via mediating cellular iron homeostasis, which is also present in stroke (Li C. et al., 2021). Mitochondrial permeability transition pore opening is a common event of ferroptosis and necroptosis, which stimulates formation of autophagosome, mitophagy, and ROS production, causing necroptosis/ferroptosis (Basit et al., 2017). Heat shock protein 90 (HSP90) is defined as a common regulatory nodal between ferroptosis and necroptosis (Wu et al., 2019). Friedmann Angeli et al. demonstrated that necrostatin-1 (RIP1 inhibitor) inhibits ferroptosis through a necroptosis/RIP1-independent manner, but ferroptosis inhibitors cannot inhibit necroptosis (Friedmann Angeli et al., 2014) and the specific link between ferroptosis and necroptosis remains to be clarified after stroke (Zille et al., 2017; Zhou et al., 2021). Carthamin yellow attenuates cerebral I/R injury via suppressing NF-κB/NLRP3-mediated pyroptosis and ACSL4-mediated ferroptosis in rats (Guo et al., 2021b). The regulatory relationship between ferroptosis and pyroptosis has been proved. Meihe et al. found that silenced or upregulated NLRP1 inflammasome positively affects ferroptosis in the oxidative stress model, and NLRP1, NLRP3, IL-1β, and caspase-1 levels are positively correlated with ferroptosis following ferroptosis inhibition or ferroptosis activation (Meihe et al., 2021). However, whether ferroptosis facilitates apoptosis, autophagy, necroptosis, pyroptosis, or other PCDs or their mutual regulation following stroke requires the further studies.

## Limitations

This is the first bibliometric analysis of “ferroptosis in stroke”, but some limitations are presented. Firstly, the retrieval time is October 30, 2021, but the database continues to be updated. Secondly, the search terms “TS=(Ferroptosis) OR TS=(Ferroptotic)”, AND “TS=(stroke) OR TS=(cerebrovascular accident) OR TS=(cerebrovascular apoplexy)”, AND Language: English, AND Reference Type: Article OR Review” are used to define the topic of this studies in the WOSCC database; some productions may not be contained. Thirdly, there are still some articles that are not included in the WOSCC database, so they are left out. However, WOSCC is the dominant database for bibliometric analysis, and we believe this work could represent the overall situation and general trend for “ferroptosis in stroke”.

## Conclusion

Our study discussed the research status of “ferroptosis in stroke” by bibliometric analysis, which is an increasingly hot research topic, with more and more scholars, institutions, and countries pouring in and publishing a lot of high-quality productions. Most of them are Chinese scholars, but most of them are isolated and lack communication and cooperation. Researchers need to strengthen the sharing of results on this topic and promote the exploration of relevant hotspots. The mechanisms of apoptosis, necroptosis, pyroptosis, and autophagy engage crosstalk with ferroptosis in the pathological processes of stroke, and the different types of PCD is as a single, but in which the individual pathway is highly interconnected and concertedly compensated for others (Bedoui et al., 2020). The ferroptosis inhibitors and targeting them are potential treatment options for stroke, but more direct clinical evidence remains to be explored. Further exploring the mechanisms of crosstalk between ferroptosis and other PCDs improves clinical applications and therapeutic effects against stroke, and a combination of ferroptosis with other harmful pathway suppression may provide valid therapies for stroke and brain disorder. The scholars will also continue to pay attention to and be interested in the hot topic of “ferroptosis in stroke”.
